# Maladaptive mother–child interactions in mothers with remitted major depression are associated with blunted amygdala responses to child affective facial expressions

**DOI:** 10.1017/S0033291724003404

**Published:** 2025-02-05

**Authors:** Catherine Hindi Attar, Neele Ridder, Jenny Stein, Dorothea Kluczniok, Katja Dittrich, Charlotte Jaite, Stephanie Spengler, Katja Bödecker, Sina Poppinga, Corinne Neukel, Judith von Schönfeld, Sabine Herpertz, Romuald Brunner, Kristina Meyer, Andreas Heinz, Felix Bermpohl

**Affiliations:** 1 Department of Psychiatry and Neurosciences, Charité Universitätsmedizin – Berlin, Corporate Member of Freie Universität Berlin and Humboldt-Universität zu Berlin, Berlin, Germany; 2 Campus St. Hedwig Hospital, Department of Psychiatry and Psychotherapy, Charité Universitätsmedizin – Berlin, Corporate Member of Freie Universität Berlin and Humboldt-Universität zu Berlin, Berlin, Germany; 3 Department of Child and Adolescent Psychiatry, Charité Universitätsmedizin – Berlin, Corporate Member of Freie Universität Berlin and Humboldt-Universität zu Berlin, Psychosomatics and Psychotherapy, Berlin, Germany; 4 Department of Clinical Psychology and Psychotherapy in Childhood and Adolescence, University of Hildesheim, Hildesheim, Germany; 5 Charité Universitätsmedizin – Berlin, Corporate Member of Freie Universität Berlin and Humboldt-Universität zu Berlin, Institute of Medical Psychology, Berlin, Germany; 6 Department for General Psychiatry, Center of Psychosocial Medicine, University of Heidelberg, Heidelberg, Germany; 7 German Center for Mental Health (DZPG), Mannheim, Germany; 8 Department of Child and Adolescent Psychiatry, Psychosomatics and Psychotherapy, University of Regensburg, Regensburg, Germany

**Keywords:** amygdala, vmPFC, insula, child affective faces, remitted depression, early life maltreatment, emotion, fMRI, maternal sensitivity, human parental brain, mother-child interactive behavior, amygdala functional connectivity

## Abstract

**Background:**

Maternal depression is associated with difficulties in understanding and adequately responding to children’s emotional signals. Consequently, the interaction between mother and child is often disturbed. However, little is known about the neural correlates of these parenting difficulties. Motivated by increasing evidence of the amygdala’s important role in mediating maternal behavior, we investigated amygdala responses to child sad and happy faces in mothers with remitted major depression disorder (rMDD) relative to healthy controls.

**Methods:**

We used the sensitivity subscale of the emotional availability scales and functional magnetic resonance imaging in 61 rMDD and 27 healthy mothers to examine the effect of maternal sensitivity on mothers’ amygdala responses to their children’s affective facial expressions.

**Results:**

For mothers with rMDD relative to controls, we observed decreased maternal sensitivity when interacting with their child. They also showed reduced amygdala responses to child affective faces that were associated with lower maternal sensitivity. Connectivity analysis revealed that this blunted amygdala response in rMDD mothers was functionally correlated with reduced activation in higher-order medial prefrontal areas.

**Conclusions:**

Our results contribute toward a better understanding of the detrimental effects of lifetime depression on maternal sensitivity and associated brain responses. By targeting region-specific neural activation patterns, these results are a first step toward improving the prediction, prevention, and treatment of depression-related negative effects on mother–child interaction.

## Introduction

The ability of a mother to understand and respond appropriately to her child’s behavioral and emotional signals is fundamental for the child’s well-being as well as for the formation of a healthy and functional mother–child relationship (Strathearn, 2011). Accordingly, emotional sensitivity has been defined as appropriate, contingent, and consistent recognition and responsiveness to infant communicative signals (Musser et al., 2012). Animal research has suggested that emotional sensitivity is causally related to the offspring’s long-term developmental outcome in several domains, including stress reactivity and maternal behavior in adulthood (Champagne et al., 2006; Maguire & Mody, 2016). In humans, studies have shown that sensitive maternal behavior was central to positive parenting and to child’s physiological, cognitive, and social–emotional development (Feldman, 2007; Feldman et al., 2004).

Findings from functional magnetic resonance imaging (fMRI) suggest that the neural correlates of maternal parenting behavior are rooted in an interconnected ‘human parental brain’ network (Swain, 2011), including different cortico-limbic brain circuits associated with a wide array of cognitive, motivational, and affective functions key to parenting (Atzil et al., 2011; Barrett & Fleming, 2011; Pereira & Ferreira, 2016). These brain circuits are partly overlapping and include those for emotion processing (amygdala, insula, anterior cingulate cortex), salience and motivation (amygdala, insula, striatum), emotion regulation (ventrolateral prefrontal cortex) as well as empathy and theory of mind circuits (medial prefrontal cortex (MPFC), tempoparietal junction, and precuneus) (Moses-Kolko et al., 2014).

The amygdala is a central node of the limbic motivational system implicated in both the maternal and affective neural networks with dense reciprocal connections to frontal control regions (Kienast et al., 2008; Phillips et al., 2003). There is profound evidence supporting its important role for stress-related components of maternal behavior and maternal–infant bonding formation (Cardinal et al., 2002). In rodent models of mothering, lesions to the amygdala or inactivation of subcomponents of the amygdala have been shown to cause severe deficits in maternal behavior (Numan et al., 2010; Toscano et al., 2009). The few human studies to directly link fMRI responses to maternal sensitivity observed during mother–child interactions provided a mixed pattern of results. Specifically, amygdala responses to infant distress stimuli (i.e. baby crying) were shown to be positively (Kim et al., 2011), negatively (Firk et al., 2018), or not at all related to maternal sensitivity (Musser et al., 2012; Olsavsky et al., 2021). Furthermore, sensitive (‘synchronous’) relative to non-sensitive mothers showed enhanced amygdala connectivity to insular, lateral, and medial frontal regions in response to infant video stimuli (Atzil et al., 2011); however, no direct maternal sensitivity effect on the amygdala *per se* was observed (Wan et al., 2014). Importantly, this research so far has relied on brain function within healthy mothers, and less attention has been paid to specific factors that may restrict a mother’s ability to respond to her child’s signals, such as a history of depression. The few existing studies on mothers with postpartum depression (PPD), which reduces maternal sensitivity (Feldman et al., 2009), have indicated an inverse relation between PPD symptom severity and amygdala responsivity to infant affective faces (Laurent & Ablow, 2013; Moses-Kolko et al., 2010). Moreover, PPD mothers showed decreased amygdala-mPFC and amygdala-insula connectivity, which were further associated with increased symptoms of depression and anxiety (Moses-Kolko et al., 2010; Wonch et al., 2016).

Here, we sought to extend the current knowledge on the neurobiological basis of maternal sensitive behavior to mothers with a former history of depression (remitted major depression disorder (rMDD)). A subgroup of the included rMDD mothers had experienced early-life maltreatment (ELM), which has been associated to reduced maternal sensitivity independently of depression (Kluczniok et al., 2016). Because ELM might thus pose an extra risk for insensitive behavior in mothers with rMDD, we sought to identify additional effects of ELM, as previously demonstrated for healthy mothers with ELM using the same face paradigm (Neukel et al., 2019). Extending previous work on mother–infant dyads with infants under 2 years, we investigated mothers of children at primary school age (5–12 years). This older age group offers a more complex interactive repertoire, which allowed us to investigate maternal sensitivity effects during mother–child interactions beyond basic aspects of caregiving behavior. During neuroimaging, we built upon previous work on maternal sensitivity in depression by using child face stimuli, which have been shown to serve as social salient stimuli especially to mothers by promoting feelings of care and empathy (Thompson-Booth et al., 2014; Zhang et al., 2020). Because we were interested in group differences in amygdala’s responsiveness to child faces across a range of affects, an affect recognition task was applied in which mothers viewed happy, sad, and neutral facial expressions of their own and an unfamiliar child. We aimed to examine three main hypotheses: First, mothers with a history of depression relative to healthy mothers were expected to show lower maternal sensitivity toward their child during mother–child interactions. This behavioral observation should be associated with decreased amygdala activation in response to child sad and happy faces (hypothesis 1). Second, mothers with a history of depression versus healthy mothers were expected to show decreased functional connectivity between amygdala and insular as well as ventromedial prefrontal regions (VMPFC) when viewing child sad and happy faces (hypothesis 2). Last, within depression groups, the subgroup of rMDD mothers with ELM was expected to show behavioral and neural response patterns that add to the effects observed for rMDD mothers without ELM (hypothesis 3).

## Methods and materials

### Sample

Eighty-eight right-handed mother–child dyads participated in this study that was approved by the ethics committee of the Charité – Universitätsmedizin Berlin (*n* = 30 rMDD mothers (rMDD), *n* = 32 rMDD mothers with an additional history of ELM (rMDD&ELM), and *n* = 26 healthy control mothers [HC]). To assess ELM, we conducted the Childhood Experience of Care and Abuse (CECA) Interview (Bifulco et al., 1994). The semi-structured clinical interview is designed to collect retrospective accounts of adverse childhood experience up to an age of 17 years, such as physical and sexual abuse, emotional abuse, neglect, and antipathy, which were rated on 4-point scales of severity (‘severe’, ‘moderate’, ‘mild’, or ‘little/none’). Mothers of the rMDD&ELM group had to score with at least moderate severity on the physical or sexual abuse scales. They had to live together with their children aged between 5 and 12 years, which already attended primary school. To assure full remission of former episodes of depression, mothers were required to have a Hamilton Depression Scale (HAMD) score below or equal to seven (Hamilton, 1960). Exclusion criteria for all mothers were neurological diseases, acute axis I disorders, a lifetime history of schizophrenia, or manic episodes as assessed by the Mini International Neuropsychiatric Interview (M.I.N.I) (Lecrubier et al., 1997), and one of the following personality disorders: emotional-unstable, anxious-avoidant, or antisocial personality disorder, based on the International Personality Disorder Examination (Loranger et al., 1997). Further exclusion criterion was the intake of benzodiazepines within the last 6 months. Medication with psychotropic drugs did not represent an exclusion criterion, however, the dosage had to be stable for at least 2 weeks prior to study entrance.

The parenting stress index (PSI) was administered to assess the magnitude of stress in the parent–child system. The PSI consists of 48 items, which comprise salient child (5 subscales) and parent characteristics (7 subscales) supposed to contribute to parental stress (Tröster, 2011). Verbal intelligence was estimated by the Wortschatz-test (Schmidt & Metzler, 1992). All participants gave written informed consent and received financial compensation for study participation.

#### Behavioral measure of maternal sensitivity

Maternal sensitivity was assessed during mother–child interactions as one out of seven scales of the Emotional Availability Scales (EASs, 4th Edition, (Biringen, 2008). Sensitivity is characterized by positive and authentic maternal affect, maternal awareness of, and responsiveness to the timing of child behavior. Low scores indicate maternal behavior that lacks general positive affect, appropriate awareness of, and responsiveness to the child’s emotional expressions. Mother–child interactions were videotaped in a standardized playroom setting. A 15-min free play condition was followed by a 6-min period in which dyads performed a highly challenging puzzle task. Maternal sensitivity was rated across the two conditions (i.e. free play and task) by an independent, trained rater team blind to maternal group membership (interrater reliability was between *r* = .78 and *r* = .86).

#### fMRI paradigm: affect recognition task

Maternal neural processing of child affective facial expressions was examined by means of an fMRI affect recognition task based on a fully crossed 3 × 2 within-subjects design with the factors affect (child happy, neutral, and sad facial expression) and identity (own or unfamiliar child facial expression). Pictures of child faces were created beforehand based on videotapes recorded during a mood induction session and rated in terms of valence by an independent, trained rater team (see (Kluczniok et al., 2017) for details). In total, 180 pictures were presented in a pseudorandomized sequence across two experimental sessions (9 min each). Of these, 90 pictures showed facial expression of the own child (OC) and 90 pictures showed faces of an age and gender matched unfamiliar control child (UC). Before scanning, subjects completed a practice session with a different set of 10 faces to ensure task comprehension.

A trial started with the presentation of the child face (2 s) followed by a fixation cross for a randomly chosen period of 2–6 s. Mothers were instructed to classify the depicted affective facial expression as quickly and accurately as possible by pressing the corresponding key on a button box with the right hand. After scanning, all faces (*n* = 180) were presented to the subjects for a second time and subjects were asked to rate each picture about valence and arousal.

#### fMRI data acquisition

Functional imaging was performed on a 3-Tesla whole-body MR scanner (Trio; Siemens) equipped with a 32-channel head coil. Thirty-three transverse slices (slice thickness, 3 mm; gap, 0.75 mm) were acquired in each volume covering the temporal lobe and occipital and orbitofrontal cortex. A T2*-sensitive gradient echo-planar imaging sequence was used (repetition time (TR) = 2 s; echotime (TE) = 2 s; flip angle = 78°; FOV = 192 × 192 mm^2^; in-plane resolution 64 × 64 mm). In addition, isotropic high-resolution (1 × 1 × 1 mm^3^) structural images were recorded using a T1-weighted coronal-oriented MPRAGE sequence with 192 slices.

### Data processing and statistical analysis

#### Demographic, behavioral, and clinical data

Comparisons of sample characteristics were conducted in SPSS version 26 (IBM corp., 2019) using a one-factor ANOVA for comparisons of all three groups (rMDD, rMDD&ELM, and HC). Maternal sensitivity was analyzed with a one-factor ANOVA with HC, rMDD, and rMDD&ELM as the group factor. The proportion of correct affect classifications of child facial expressions during the fMRI task as well as the post-scan valence and arousal ratings on these pictures were analyzed using 3 × 3 × 2 ANOVAs with HC, rMDD, and rMDD&ELM as the group factor, and child affect (happy, neutral, sad) and identity (familiar, unfamiliar) as within-subjects factors. Where appropriate, the Huynh–Feldt procedure was applied to correct for violations of the sphericity assumption and a two-tailed approach to significance was used for the statistical tests.

#### fMRI data processing

Functional imaging data were analyzed using SPM12 (Welcome Department of Cognitive Neurology, London) and custom scripts in MATLAB (version 2018a, The MathWorks). For preprocessing information, see Supplementary Material. Subject-level models included separate regressors for all six combinations of child facial expressions (sad, happy, neutral) and identity (own, unfamiliar) in each session and regressors were convolved with the hemodynamic response function. Regressors were subjected to a second-level whole-brain analysis of variance (flexible factorial) with all six conditions (i.e. sad, happy, and neutral facial expressions of the own and an unfamiliar child). Although identity conditions (own or unfamiliar child face) were modelled separately, the main goal of the present study was to test group differences between depressed (rMDD, rMDD&ELM) and non-depressed mothers (HC). Thus, we first performed group by identity interaction contrasts by analyzing brain responses to emotional expressions of own and unfamiliar child facial expressions. We failed to detect any significant group by identity interaction effects; thus, for the following main analyses, group by facial affect contrasts were always calculated across the factor identity (i.e. by equally addressing facial expressions of the own and unfamiliar child).

### fMRI data analysis

We tested group by affect interaction differences in amygdala responsivity and connectivity using a region of interest (ROI) analysis approach. For the amygdala and insular regions, mask images taken from the FSL Harvard-Oxford structures atlas (thresholded at 50% probability, Harvard Center for Morphometric Analysis; https://fsl.fmrib.ox.ac.uk/fsl/fslwiki/Atlases). For the vmPFC we used – analogue to previous work (Tiedemann et al., 2020) – a sphere with 20 mm radius that was centered on the peak voxel (2, 46, 8) derived from 199 imaging studies reporting ‘vmPFC’, as determined by a meta-analysis conducted on the www.neurosynth.org platform. To explore neural effects outside these ROI analyses, they were followed by whole-brain analyses using a more lenient threshold of *P*_uncorr_ < .001.

To test hypothesis 1 stating decreased amygdala activation in response to child affective faces for remitted depressed relative to healthy mothers, the group by facial affect contrasts (rMDD+rMDD&ELM < HC: own+unfamiliar child sad, happy, neutral faces) were entered into the flexible factorial model. To investigate the association between amygdala responses to child affective faces and maternal sensitivity, we performed regression analyses across groups within SPM in which mothers’ individual scores of maternal sensitivity from the mother–child interaction were correlated with their neural responses to child affective faces using the group factor as covariate of no interest.

To test hypothesis 2 stating decreased functional connectivity between amygdala (seed region) and insular as well as medial frontal regions in response to child affective facial expressions for remitted depressed relative to healthy mothers, we used the generalized form of psychophysiological interaction analysis (gPPI) (McLaren et al., 2012). Therefore, amygdala time series derived from the group comparison analysis done for hypothesis 1 were extracted and PPI regressors for task × time series interactions were entered into a flexible factorial model using the between-subject factor group (rMDD+rMDD&ELM, HC) and the within-subject factor child facial affect (sad, happy).

Finally, to test the hypothesized additional effect of ELM on maternal behavioral and neural responses (hypothesis 3), the contrast rMDD&ELM > rMDD: own+unfamiliar child sad, happy, neutral faces was entered into the flexible factorial model also used for group comparisons associated with hypothesis 1.

## Results

### Sample characteristics and fMRI task performance

As given in Table 1, maternal groups did not differ in terms of age, IQ, and years of education. We found significant group differences for mothers with a history of depression and healthy mothers in the HAMD depression scores. However, since we only included mothers with depression in remission, HAMD scores in both depression groups were still well below the clinically relevant level. Groups also differed with respect to partnership status with a higher number of mothers with a history of depression being separated, divorced, and single (single status differed only for the rMDD&ELM mothers from HCs) and a lower amount of rMDD mothers being married. We further observed a significant main effect group on the perceived level of parenting stress (PSI), with significant differences between parenting stress scores of rMDD mothers (rMDD, rMDD&ELM) relative to healthy mothers while mothers with rMDD and ELM did not differ from rMDD mothers without ELM.

**Table 1.** Sample characteristics

	HC	rMDD	rMDD & ELM	*P*-value
	(*n* = 26)	(*n* = 30)	(*n* = 39)	
Age (mean, SD)	39.3 (5.7)	39.5 (5.8)	40.2 (7.6)	>.05
IQ (mean, SD)	109.4 (10.2)	105.9 (11.4)	106.8 (9.4)	>.05
Education in years (mean, SD)	17.7 (3.0)	16.7 (3.7)	17.2 (4.3)	>.05
Working (%)	92.3	72.4	74.2	>.05
Partnership status (%)				
Single	3.8	3.4	12.9	<.05
Married	84.6	48.3	58.1	<.05
Separated/divorced	11.5	48.3	25.8	<.01
HAMD (mean, SD)	0.7^a^ (1.1)	2.7^b^ (2.0)	2.3^b^ (2.0)	<.0001
PSI (mean, SD)	68.8^a^ (21.6)	84.1^b^ (20.5)	80.1^b^ (16.4)	<.05

*Note:* ELM, early-life maltreatment; HAMD, Hamilton rating scale for depression; HC, healthy controls; IQ: intelligence quotient, measured by a German vocabulary test (Wortschatztest, WST); *n*, subsample size; PSI, parenting stress index; rMDD, mothers with depression in remission.

^a,b^Superscript letters represent Bonferroni-corrected post-hoc tests. Two values with the same letter are not significantly different from each other.

Finally, at the time of study inclusion, the patient groups were characterized by only *n* = 1 having the comorbid diagnosis of an eating disorder.

Affect recognition performance during the fMRI task as well as post-scan valence and arousal ratings of the child face stimuli did not show further differences between groups (for further sample characteristics on fMRI task performance and post-scan ratings see Supplementary Material).

### Associations between maternal sensitivity and amygdala activation

#### Maternal sensitivity

**Figure 1.**
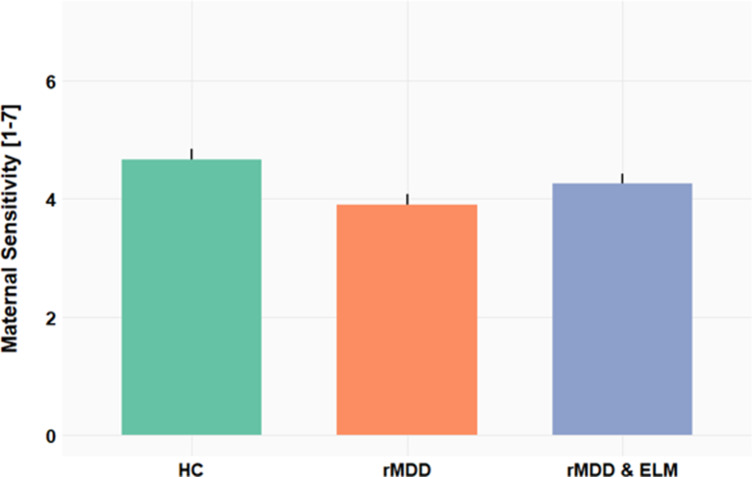
Maternal sensitivity scores yielded a main effect of group with mothers with remitted depression (rMDD, rMDD&ELM) showing lower maternal sensitivity compared to healthy control mothers (HC).

We observed a significant main effect of group on mothers’ sensitivity in response to their child (*F*(2,82) = 4.17, *P* < .05, Figure 1). Tukey post-hoc analysis revealed a significant difference (*P*< .05) between mean maternal sensitivity scores of the two groups of rMDD mothers (rMDD, rMDD& ELM) relative to healthy mothers (−.7634, 95%-CI [−1.395 −.132]).

#### Amygdala responsivity

**Figure 2.**
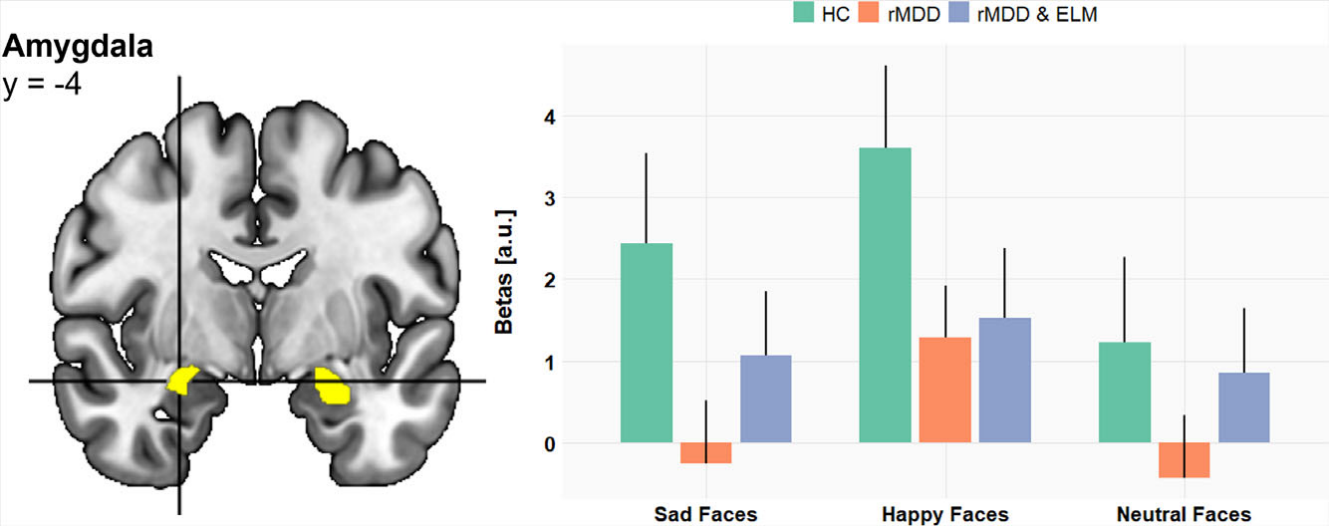
Group comparison showing for mothers with remitted depression (rMDD, rMDD&ELM) reduced left and right amygdala responses to sad, happy, and neutral facial expressions. Parameter estimates of the right amygdala peak voxel (28, −4, −14) are displayed on the right-hand side (error bars reflect standard error of the mean).

Mothers with a history of depression (rMDD, rMDD&ELM) relative to HC, showed reduced bilateral amygdala responses to child sad (−24, −12, −12, *T* = 4.65, ROI *P*_FWE_ = .0001; 28, −4, −14, *T* = 5.96, *P*_FWE_ < .0001), happy (−20, −4, −12, *T* = 2.97, ROI *P*_FWE_ = .051; 26, −6, −12, T = 6.43, ROI *P*_FWE_< .0001), and neutral facial expressions (−18, −8, −12, *T* = 3.14, ROI *P*_FWE_ < .05; 26, −6, −12, *T* = 3.53, ROI *P*_FWE_< .05) (Figure 2).

#### Brain–behavior correlation

**Figure 3.**
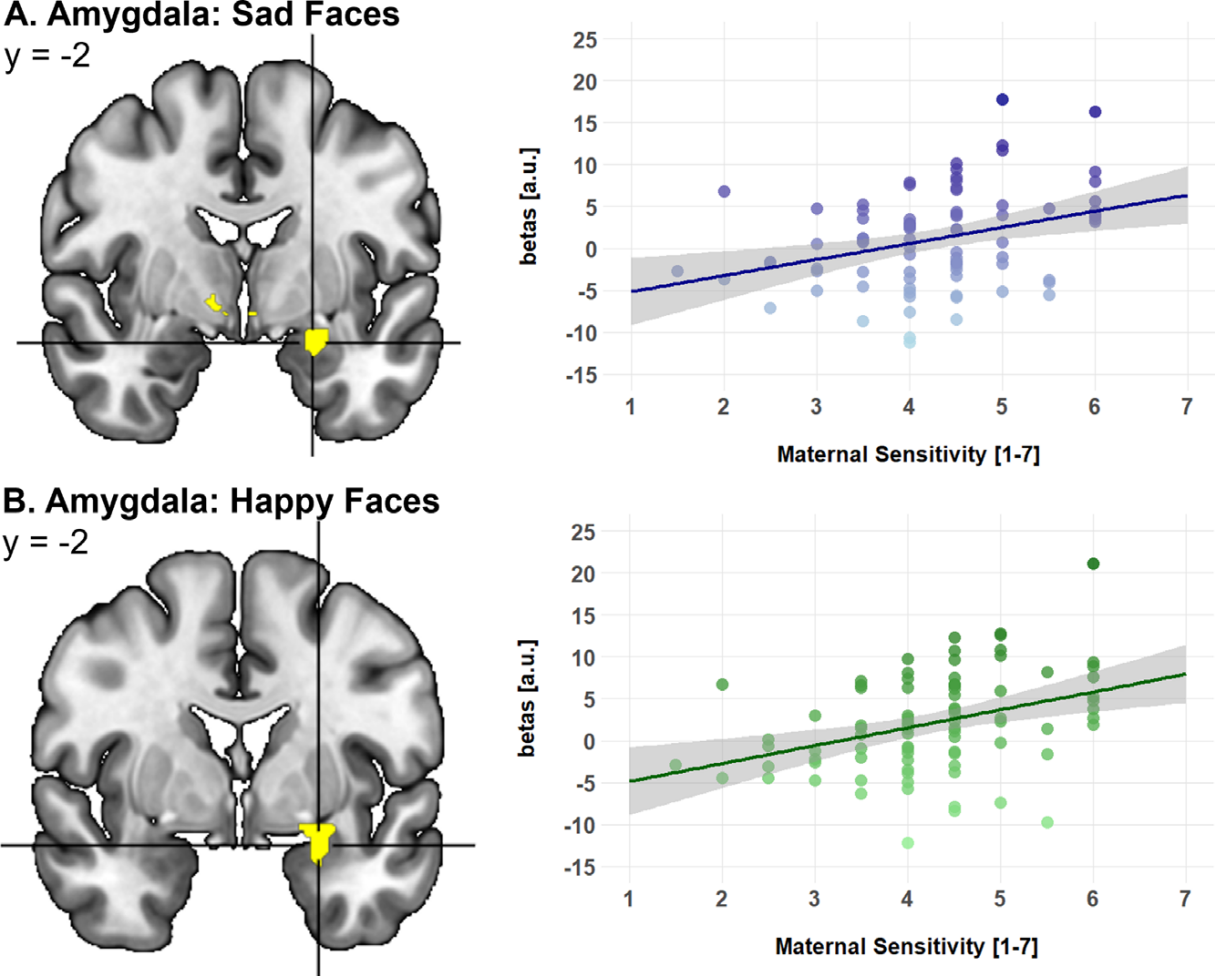
Maternal sensitivity in association with maternal neural responses to (a) sad and (b) happy child facial expressions. For visual purpose, the statistical maps are displayed at *P* < .001 uncorrected across the whole brain. Scatter plots illustrating the positive correlations between amygdala responses to sad and happy faces between the percent signal change in the left amygdala and the degree of maternal sensitivity based on observations during mother–child interactions (higher values represent higher degrees of maternal sensitivity).

To test whether the reduced maternal sensitivity and the reduced amygdala responses observed for the rMDD mothers with and without ELM were related to each other, a regression analysis conducted within SPM 12 across all three groups of mothers (controlling for the diagnostic group) was done. This analysis yielded a strong positive association of maternal sensitivity and left amygdala responsiveness to child facial affective expressions. Specifically, as shown in Figure 3, higher behavioral scores of maternal sensitivity were significantly associated with increased amygdala responses to sad (−24, −2, −18, *T* = 3.22, ROI *P*_FWE_ < .05) and happy child faces (−24, *y* = −2, *z* = −18, *T* = 3.68, ROI *P*_FWE_ < .05, Figure 3). Interestingly, we found no significant relation between maternal sensitivity and mothers’ amygdala responses to neutral faces.

In sum and confirming hypothesis 1, we found rMDD relative to healthy mothers to show reduced maternal sensitivity during mother–child interactions, reduced amygdala responses to child sad, happy, and neutral faces and we observed a significant association between maternal sensitivity and amygdala responsiveness to child sad and happy faces.

### Fronto-limbic functional connectivity

**Figure 4.**
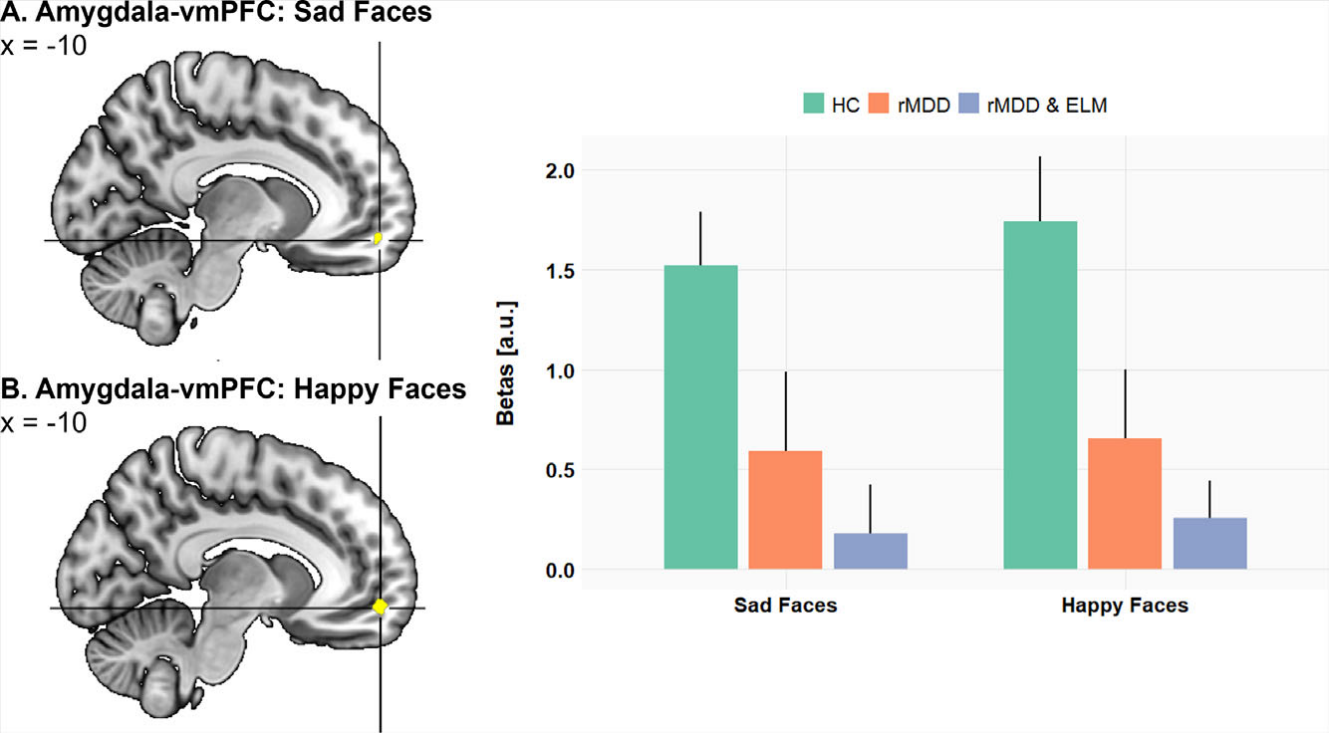
Functional connectivity analysis from left and right amygdala as seed regions: In response to (a) sad child faces and (b) happy child faces, mothers with remitted depression (rMDD, rMDD&ELM) relative to healthy control mothers (HC) show significantly lower functional correlation between the left and right amygdala region with the ventromedial prefrontal cortex (vmPFC). Parameter estimates of the amygdala connectivity with the vmPFC peak voxel (−10, 56, −12) are displayed on the right-hand side (error bars reflect standard error of the mean).

**Figure 5.**
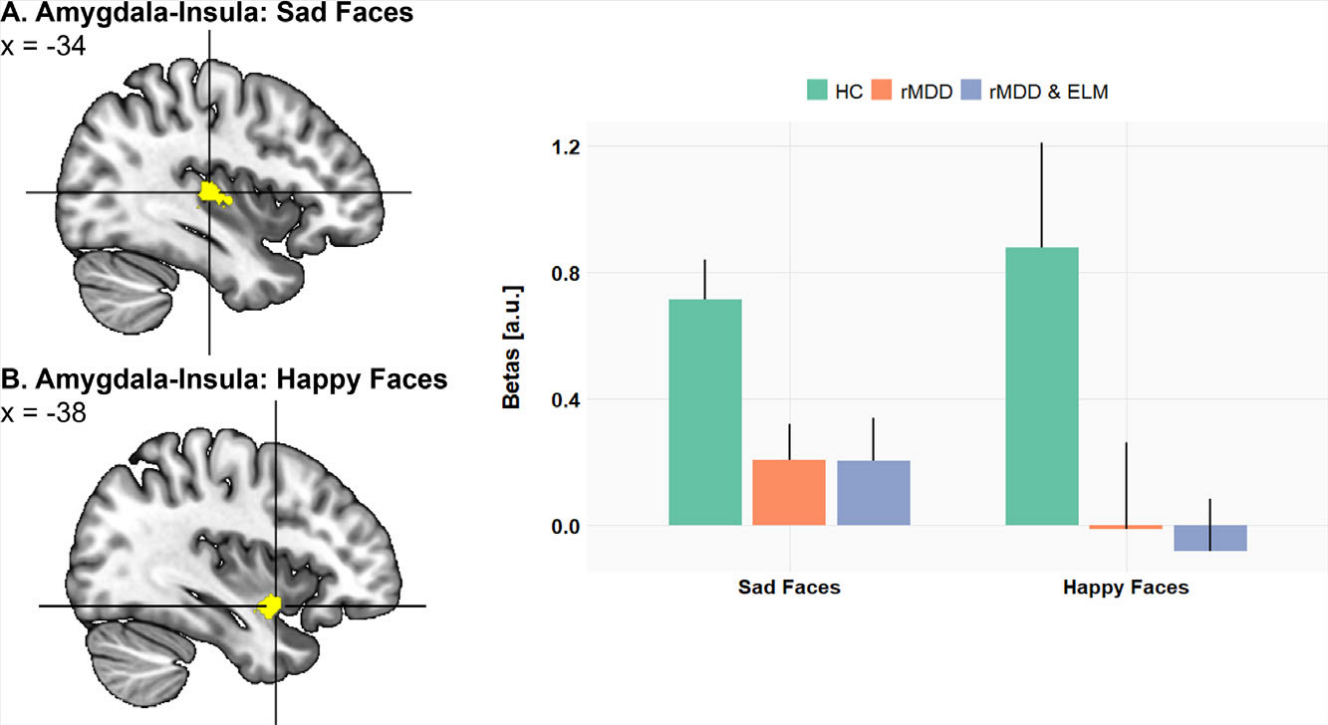
Functional connectivity analysis from left and right amygdala as seed regions: In response to (a) sad child faces and (b) happy child faces, mothers with remitted depression (rMDD, rMDD&ELM) relative to healthy control mothers (HC) show significantly lower functional correlation between the left and right amygdala region with the left insula. Parameter estimates of the amygdala connectivity with the insula peak voxel (−38 2 −12) are displayed on the right-hand side (error bars reflect standard error of the mean).

We next investigated the effect of remitted maternal depression on the coupling of the amygdala with other brain regions, especially the ventromedial prefrontal cortex (vmPFC) and the insula. Confirming hypothesis 2, mothers with a history of depression (rMDD, rMDD&ELM) relative to healthy mothers showed reduced functional connectivity of left and right amygdala seed regions with the vmPFC during the processing of sad (left amy-vmPFC: −10, 56, −12, *T* = 4.17, ROI *P*_FWE_< .05; right amy-vmPFC: −10, 56, −10, *T* = 4.30, ROI *P*_FWE_< .05) and happy child faces (left amy-vmPFC: −12, 56, −10, *T* = 4.46, ROI *P*_FWE_< .01; right amy-vmPFC: −12, 56, −10, *T* = 5.96, ROI *P*_FWE_< .0001, Figure 4). Similarly, we also observed reduced amygdala-insula coupling for mothers with remitted depression (rMDD, rMDD&ELM) relative to healthy mothers in response to sad (left amy-left insula: −34, −20, 8, *T* = 4.25, ROI *P*_FWE_ < .05) and happy child faces (left amy-left insula: −38, 2, −12, *T* = 4.66, ROI *P*_FWE_ < .005, right amy-left insula: −40, 2, −12, *T* = 4.16, ROI *P*_FWE_ < .05, Figure 5).

### Effects of early life maltreatment

To test whether ELM had a separate effect on the amygdala activation and connectivity, we compared rMDD mothers with ELM with rMDD mothers without ELM experience. Behaviorally, we did not observe an additional effect of ELM on maternal sensitivity scores during mother–child interactions: there was no significant difference (*P* > .05) between mothers with rMDD and ELM and rMDD mothers without ELM (−.3615, 95%-CI [−.236 .959]).

Similarly, at the neural level, rMDD mothers with ELM vs. rMDD mothers without ELM did not differ significantly in their amygdala activation and amygdala functional connectivity in response to sad, happy, and and neutral child facial expressions. To further control for any effects of ELM on amygdala activation in response to sad and happy faces, we performed an additional regression analysis within SPM 12 across all three groups of mothers including the regressor ELM. This analysis did not yield any significant ELM effect on amygdala responsiveness to child facial affective expressions.

Thus, hypothesis 3 stating an additional effect of ELM on behavioral and neural indicators of maternal sensitivity could not be confirmed by the present data.

## Discussion

We investigated the effect of depression history on the association of maternal sensitivity with the amygdala responsiveness to child affective faces. Mothers with a history of depression relative to healthy mothers showed decreased maternal sensitivity when interacting with their child. Importantly, this group of mothers also showed reduced amygdala responses to child neutral, sad, and happy faces and lower maternal sensitivity was associated with lower amygdala responses to child happy and sad facial expressions. Thus, we could confirm all parts of hypothesis 1 by the present data. Second and in line with hypothesis 2, we found for mothers with a history of depression relative to healthy mothers reduced amygdala-vmPFC and reduced amygdala-insula functional connectivity in response to child sad and happy faces. Finally, we predicted a specific (additional) effect of ELM experience within the group of rMDD mothers which was not confirmed by the present data so that hypothesis 3 had to be rejected.

**Figure 6.**
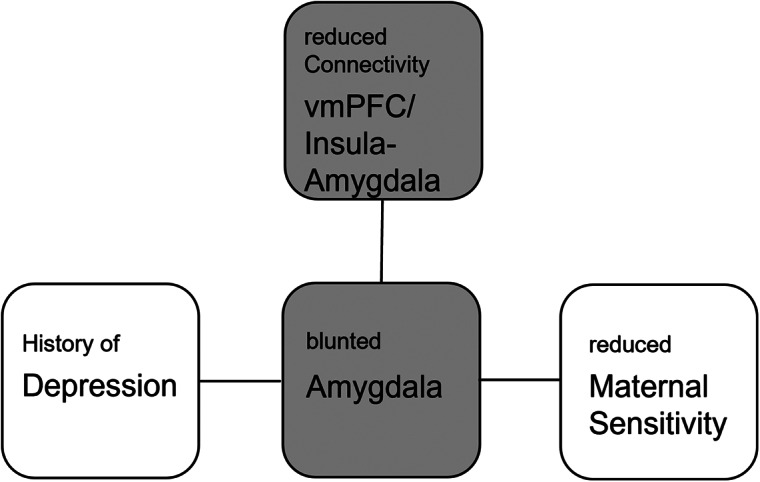
Schematic representation of a putative neurobehavioral model of maternal sensitivity. A history of depression attenuates amygdala responses to sad and happy child faces, possibly through aberrant top-down and bottom-up connectivity of the amygdala with insula and ventromedial prefrontal cortex (vmPFC), leading to reduced maternal sensitivity during mother–child interactions.

Altogether, our findings strongly suggest that blunted amygdala activation and connectivity in response to child’s affective facial expressions may serve as a putative neural correlate through which depression is associated with reduced maternal sensitivity (Figure 6).

### Maternal sensitivity in remitted depression

Our finding of reduced maternal sensitivity in rMDD mothers during mother–child interactions supports the few existing previous reports on mothers suffering from postpartum depression (Laurent & Ablow, 2013; Moses-Kolko et al., 2010; Stanley et al., 2004). However, the neural pathways underlying maternal sensitivity in depression are to date not well understood and this is the first study reporting reduced maternal sensitivity in association with blunted amygdala responsiveness in rMDD relative to healthy mothers. Importantly, our depression sample only included mothers with a history of depression well beyond the postpartum period. Thus, the present finding was shown in a sample of mothers with rMDD, which was free from hormonal changes typically associated with PPD or active depressive symptoms which likely influence the function of key brain areas underlying sensitive maternal behavior (Barrett & Fleming, 2011). Therefore, reduced maternal sensitivity and amygdala responses in rMDD mothers are likely to be long-term consequences of the depression as illustrated in our model of maternal sensitivity pathways (Figure 6).

Notably, we observed a positive association between maternal sensitivity and amygdala responses to both child sad and happy facial expressions. Most of the few previous studies linking maternal parenting behavior to amygdala responsiveness were using infant cry stimuli (Atzil et al., 2011; Firk et al., 2018; Kim et al., 2011; Musser et al., 2012; Olsavsky et al., 2021; Wan et al., 2014). The previously observed maternal neural responses were thus restricted to infant distress situations and could either reflect specific behaviors of care in mothers, such as the urgency to understand and reduce the infant stress or they could reflect mothers’ own distress in reaction to cry. In depressed mothers, Laurent and colleagues (Laurent & Ablow, 2013) reported blunted neural responses in self-regulatory and motivational neural circuits to both own infant distress and own infant joy faces. Our finding of similarly blunted amygdala responses to child happy and sad faces together with the observed strong association between maternal sensitivity and amygdala responsiveness to child affective faces thus emphasizes the major role of the amygdala in detecting salient information across a range of affects to guide positive, pro-active, and pro-social maternal behaviors, which are key aspects of healthy parenting (Laurent & Ablow, 2012; Moses-Kolko et al., 2010).

### Amygdala functional connectivity

Although evidence has accumulated in support of the amygdala as a critical structure for maternal sensitivity, it is likely not the only brain region to explain maternal behavior. Our finding of decreased functional connectivity between the amygdala and the vmPFC as well as the insula in response to child sad and happy faces in mothers with rMDD is in good accordance with previous reports of fronto-limbic hypoconnectivity in women with postpartum depression in response to positive infant faces (Wonch et al., 2016).

The insula which is associated with the Theory of Mind network, is known to represent the physiological state of the body, a necessary prerequisite to guide subjective emotional experience (Craig, 2002) with functional interconnections to regions associated with emotion experience and cognitive control (Wager et al., 2008). In this vein, previous studies have demonstrated that the connectivity of the amygdala to frontal and insular areas is involved in emotion regulation processes (Price, 2003) pointing toward a lack of top-down emotional regulation of negative emotions (Moses-Kolko et al., 2010) and reduced processing of emotionally relevant stimuli among depressed mothers (Wonch et al., 2016), both critical elements contributing to sensitive early parenting (Dayton et al., 2016).

The present finding of reduced coupling between amygdala and insular as well as medial prefrontal regions in combination with blunted amygdala responses to sad and happy faces in rMDD mothers is compatible with reduced facilitatory top-down signals from insular and medial prefrontal areas to the amygdala, which may have influenced motivational aspects of affective processing (i.e., directing attention to the child, charging the affective value). In addition, reduced bottom-up signals from the amygdala to the vmPFC via the insular hub station may have lessened higher-order mental processes. As illustrated in Figure 6, these top-down processes to the amygdala and bottom-up processes from the amygdala to medial frontal regions might support adequate representations of child’s needs and adaptive theory of mind skills, all of which are mandatory prerequisites of emotional sensitive parenting (Swain, 2011).

### Effects of ELM in remitted depression

For rMDD mothers with ELM relative to rMDD mothers without ELM, we did not observe a specific (additive) effect of ELM on maternal sensitivity and neural responses to child facial affective expressions. This was unexpected, as previous studies associated ELM with higher amygdala responsiveness to negative face stimuli (Dannlowski et al., 2012). Several reasons may explain this lack of ELM effect: First, our sample differs from others as the included mothers with ELM all had a history of depression. Thus, the depression effect could have overwritten the effect of trauma. Second, there is recent evidence for specific sensitive periods in which exposure to ELM leads to the most significant neural alterations. Specifically, it has been shown that amygdala responses to threat stimuli were blunted for prepubertal but enhanced for postpubertal exposure to childhood maltreatment (Zhu et al., 2019). Because ELM in the present study was assessed across the entire childhood period up to 18 years, we could not verify similar timing effects. Finally, to clearly disentangle the effect of ELM from depression, it would have been necessary to introduce a fourth group of healthy mothers with ELM. However, recent work comparing healthy mothers with vs. without ELM using the same affect recognition paradigm reported ELM effects in response to the own child’s happy face in mentalizing, mirror neuron, and visual processing circuits (Neukel et al., 2019). The fact that they did not observe any ELM effects in emotion processing areas and in particular in the amygdala strengthens the assumption that our observed group effect is related to depression.

### Limitations

Several limitations should be noted. First, future investigations of further aspects of emotional availability besides maternal sensitivity (i.e. structuring, nonintrusiveness and nonhostility) will be necessary to inform the specificity of our results to the neural pathway of maternal sensitivity and depression history. Second, our cross-sectional study does not allow inferring causality, only relationships between brain and behavior. To disentangle preexisting alterations from consequences of depression, longitudinal investigations of maternal sensitivity are needed. Third, in light of recent reports on the lack of reliability in small-sized neuroimaging studies linking brain function and behavior (Marek et al., 2022), the present finding of an association between amygdala function and maternal sensitivity has to be marked as preliminary. Although sample sizes in clinical studies cannot be unlimitedly enlarged, future studies using higher sample sizes, other psychiatric disorders, and variants of behavioral observations of emotional sensitivity are needed to strengthen the link between emotional sensitivity and limbic function. Finally, combinations of fMRI and behavioral assessments of parenting behavior with neurobiological markers of stress (cortisol) and attachment (oxytocin) may further enhance our understanding of specific high-risk conditions for dysfunctional parenting.

## Conclusion

To conclude, mothers with remitted depression relative to healthy mothers showed reduced maternal sensitivity while interacting with their child, which was associated with reduced amygdala responsivity to child happy and sad facial expressions. The present study thus provides strong support for the view that attenuated neural responses of the amygdala to child affective faces might serve as a potential correlate through which depression compromises and influences maternal sensitive parenting behavior.

## Supporting information

Hindi Attar et al. supplementary material Hindi Attar et al. supplementary material

## Supplementary material

The supplementary material for this article can be found at http://doi.org/10.1017/S0033291724003404.
