# Supplementary material for: Maladaptive mother–child interactions in mothers with remitted major depression are associated with blunted amygdala responses to child affective facial expressions
Source: Psychol Med. 2025 Feb 5;55:e15. doi: 10.1017/S0033291724003404 (PMC11968120; doi:10.1017/S0033291724003404)
Supplement: Hindi Attar et al. supplementary material [file S0033291724003404sup001.docx]

**Supplement**

**FMRI preprocessing analysis**

The affect recognition task was scanned in two sessions and for each session participant’s data were slice-time corrected and realigned with unwarping. The high-resolution T1 image was then coregistered with the mean EPI image and subsequently segmented using the unified segmentation approach as implemented in the “New Segment” routine in SPM8. During this latter preprocessing step, tissue-class images for gray and white matter were generated and used within the DARTEL toolbox to create structural templates across subjects as well as individual flow fields. These flow fields were then used to spatially normalize the EPI images into MNI space. Images were saved with a spatial resolution of 2 × 2 × 2 mm3, and smoothing was accomplished using an 8-mm full-width at half maximum (FWHM) isotropic Gaussian kernel. Finally, images were high-pass filtered at 128s, and an autoregressive AR(1) model was used to account for serial correlations in fMRI time series.

**FMRI Task: Affect Recognition**

Correctly identifying child facial expressions revealed a significant main effect of child affect. Across groups, happy relative to neutral or sad facial expressions were significantly better recognized, irrespective of child identity (F(2,170) = 65.4; p<.0001). No significant differences between groups were observed (Table S1).

- *Insert Table S1 about here -*

**Behavioral Valence and Arousal Rating Data**

For mother’s **valence** **ratings**, significant main effects were found for the two factors child affect (F(2,160)=842.41; p<.0001) and identity (F(1,80) =11.38; p=.001). Mothers rated happy facial expressions as most pleasant and sad expressions as most unpleasant. In addition, they rated facial expressions of their own child generally higher compared to facial expressions of the unfamiliar child. We also found a significant child affect x identity interaction effect (F(2,2) =30.77: p=.000), with a stronger identity effect for happy faces compared to neutral and sad faces. Last, a significant three way interaction child affect x identity x group (F(4,160)=2.86; p<.05) indicated that healthy control mothers rated sad facial expressions of their own child as particularly unpleasant (see also Table S2).

**Arousal ratings** revealed significant effects of “affect” (F(2,160)=77.88; p=.000) and “identity” (F(1,80) =78.99; p=.000). Specifically, mothers rated happy facial expressions as most intense compared to neutral and sad facial expressions. In addition, mothers rated facial expressions of their own child as more intense compared to facial expressions of the unfamiliar child. The significant interaction between the two factors (F(2,2) =15.90, p=.000) indicated a stronger identity effect for happy compared to neutral and sad face conditions. A three way interaction between the factors “affect”, “identity” and “group” was significant at trend level (F(4,160)=2.15; p=.077), showing that healthy mothers perceived sad facial expressions of their own child as particularly intense (see also Table S2).

- *Insert Table S2 about here -*

| **Table S1.** Affect Recognition Performance | | | |  |  |
| --- | --- | --- | --- | --- | --- |
|  |  |  |  |  |  |
|  |  | **HC** | **rMDD** | **rMDD &** | **p** |
|  |  | (n=26) | (n=30) | **ELM** (n=39) |  |
| **Own Child** | sad | 88.5 (14.8) | 78.8 (20.6) | 87.6 (15.3) | p>.05 |
| (Mean, SD) | happy | 99.7 (0.9) | 97.6 (8.6) | 99.4 (2.1) | p>.05 |
|  | neutral | 80.5 (15.7) | 78.4 (14.8) | 83.4 (15.8) | p>.05 |
| **Unfamiliar Child** | sad | 88.2 (12.6) | 79.9 (22.7) | 81.1 (24.3) | p>.05 |
| (Mean, SD) | happy | 97.9 (2.4) | 96.6 (8.6) | 98.3 (2.7) | p>.05 |
|  | neutral | 82.8 (15.3) | 83.1 (16.3) | 81.7 (16.7) | p>.05 |
| HC: Healthy Controls; rMDD: Mothers with depression in remission; ELM: Early Life Maltreatment. | | | | |  |

| **Table S2.** Behavioral Rating Scores | | | |  |  |  |
| --- | --- | --- | --- | --- | --- | --- |
|  |  |  |  |  |  |  |
|  |  |  | **HC** | **rMDD** | **rMDD &** | **p** |
|  |  |  | (n=26) | (n=30) | **ELM** (n=39) |  |
| **Valence** | **Own Child** | sad | 2.6 (0.6) | 2.8 (0.8) | 2.8 (0.8) | p>.05 |
| (Mean, SD) |  | happy | 6.6 (0.3) | 6.6 (1.5) | 6.6 (0.3) | p>.05 |
|  |  | neutral | 4.1 (0.6) | 4.2 (1.2) | 4.1 (0.5) | p>.05 |
|  | **Unfamiliar Child** | sad | 2.9 (0.5) | 2.9 (0.9) | 3.0 (0.6) | p>.05 |
|  |  | happy | 5.8 (0.6) | 6.3 (1.5) | 5.8 (0.6) | p>.05 |
|  |  | neutral | 4.1 (0.4) | 4.2 (1.1) | 4.2 (0.3) | p>.05 |
| **Arousal** | **Own Child** | sad | 5.8 (1.0) | 5.7 (2.2) | 5.9 (0.8) | p>.05 |
| (Mean, SD) |  | happy | 4.2 (0.7) | 4.2 (1.6) | 4.1 (0.6) | p>.05 |
|  |  | neutral | 5.1 (0.9) | 4.9 (1.9) | 4.8 (1.0) | p>.05 |
|  | **Unfamiliar Child** | sad | 4.8 (0.9) | 4.9 (1.8) | 4.7 (1.1) | p>.05 |
|  |  | happy | 3.7 (0.9) | 3.8 (1.3) | 3.7 (0.8) | p>.05 |
|  |  | neutral | 4.3 (0.8) | 4.5 (1.4) | 4.3 (0.8) | p>.05 |
| HC: Healthy Controls; rMDD: Mothers with depression in remission; ELM: Early Life Maltreatment. | | | | | |  |
